# Comparison of 2 different fluoroscopy activation intervals in shock wave lithotripsy: a prospective randomized study

**DOI:** 10.20452/wiitm.2025.17947

**Published:** 2025-04-17

**Authors:** Cengiz Çanakcı, Ahmet Şahan, Orkunt Özkaptan, Erdinç Dinçer, Utku Can, Alper Coşkun

**Affiliations:** Department of Urology, Health Sciences University, Kartal Dr. Lutfi Kirdar City Hospital, Istanbul, Turkey; Department of Urology, Medicana Hospital, Bursa, Turkey

**Keywords:** fluoroscopy, kidney stone, radiation, shock wave lithotripsy

## Abstract

**INTRODUCTION:**

Intermittent fluoroscopic controls are required during shock wave lithotripsy (SWL) to readjust the probe due to patients’ movements, respiratory movement, and stone displacement within the kidney. However, there is still no consensus in the literature on the optimal frequency of fluoroscopic monitoring.

**AIM:**

Our aim was to determine the optimal fluoroscopy activation interval in fluoroscopy‑guided SWL and examine its effect on fluoroscopy time and stone‑free status.

**MATERIALS AND METHODS:**

This prospective randomized study included patients with opaque renal pelvic stones smaller than 2 cm, subjected to fluoroscopy‑guided SWL between July 2020 and January 2024. The patients were divided into 2 groups. Fluoroscopic control was performed every 250 shocks in group 1, and every 500 shocks in group 2. Demographic data, calculus volume and density, skin‑to‑stone distance, number of shots and sessions, fluoroscopy duration, and stone‑free status were analyzed.

**RESULTS:**

The data of 158 randomly included patients (equally divided between both groups) were analyzed. No differences were observed between the groups in terms of demographic data and stone parameters. However, there was a difference in fluoroscopy time, which was longer in group 1 than in group 2 (mean [SD], 217.9 [90.2] vs 117 [37] s, respectively; P <0.001). No differences in stone‑free status between the groups were observed (group 1; 64.5%; group 2, 67%; P = 0.87).

**CONCLUSIONS:**

Reducing fluoroscopy activation interval in SWL does not affect stone‑free status, but it helps limit radiation exposure.

## INTRODUCTION

Shock wave lithotripsy (SWL) is a noninvasive method, widely used as the first step in treating stone disease. It is recommended as a first-line treatment for kidney stones smaller than 2 cm, due to its low complication rate and lack of anesthetic requirements.^1^^,^^2^^,^^3^ The success of SWL is affected by various factors, including the location, density, and size of the calculus, the number and frequency of shocks, and the power of the shock wave used during sessions.^4^ SWL focuses on the calculus using ultrasonography or fluoroscopy to transmit high-energy shock waves from the lithotripter to the calculus in order to fragment it.^5^ Fluoroscopic focusing is a commonly used imaging technique, but it raises concerns about ionizing radiation exposure of patients, technicians, and physicians. Minimizing or optimizing radiation exposure is crucial, especially for technicians and physicians who experience it throughout their working lives.

Ultrasound-guided focusing provides an alternative to fluoroscopy. However, it carries certain limitations: it restricts probe movement, it is difficult to perform when the stone coincides with the costal plane, and it cannot be used for ureteral stones, although it is more commonly used for the fragmentation of nonopaque stones.^6^^,^^7^^,^^8^ Intermittent fluoroscopic controls are required during SWL to readjust the probe due to patients’ movements, respiratory movement, and stone displacement within the kidney. However, there is no consensus in the literature on the optimal frequency of fluoroscopic monitoring. In our center, fluoroscopy control is regularly performed every 250 shocks. Establishing an optimal time frame for fluoroscopic monitoring might be beneficial in reducing radiation exposure.

**FIGURE 1:**
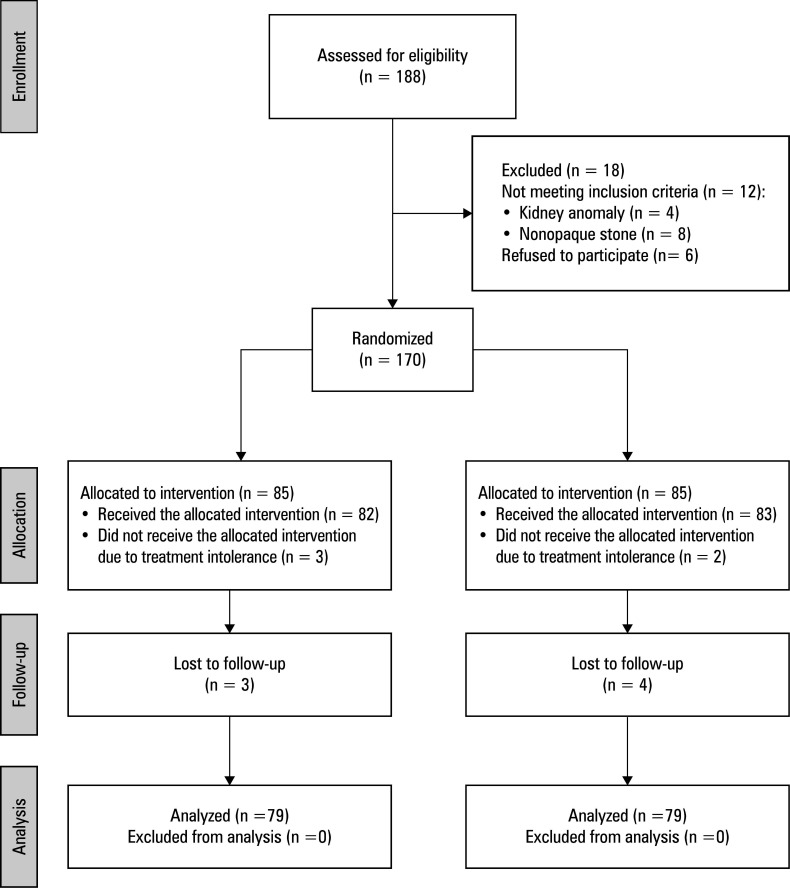
Consolidated Standards of Reporting Trials flow chart for study participants

## AIM

This study aimed to determine if increasing the time between fluoroscopic controls affected the SWL success rate in obtaining stone-free status, while reducing radiation exposure.

## MATERIALS AND METHODS

The study includ‑ ed 158 patients with radiopaque renal pelvic stones smaller than 2 cm, who underwent SWL between July 2020 and January 2024. Stone vol‑ ume was calculated using the following formula: V = π/6 × A × B × C.^9^ Patients were randomized into 2 groups. Group 1 was fluoroscopically monitored every 250 shocks, and group 2, every 500 shocks FIGURE 1. At each fluoroscopic control, stone fo‑ cusing was performed under continuous fluoro‑ scopic guidance. Only pure X‑ray equipment was used for targeting, without the ultrasound focus‑ ing system. An electromagnetic shock wave gener‑ ating device (Dornier Compact Sigma, Med Tech, Wessling, Germany) was used during the proce-dure. Prior to the commencement of the study, a statistical power analysis was conducted. A pre-liminary study of 20 cases indicated a reduction in fluoroscopy time of over 20%, with similar stone-free rates, in comparison with our retro-spective data. In a power analysis based on mean (SD) fluoroscopy time of 107 (36) seconds with a power of 90% (α = 0.05; β = 0.1), 78 patients needed to be included in each group. In order to ensure sufficient statistical power, a minimum of 170 patients had to be enrolled in the study, as-suming a 10% loss to follow-up. In our final anal-ysis, each group included 79 patients, with a pow-er of 90%. The primary end point was to evalu-ate the effect of 2 different fluoroscopic activa-tion intervals on the stone-free rate. Secondary end points were the radiation dose received and complications (such as colic pain, hematuria, py-elonephritis, and steinstrasse formation). Data on complete blood count, coagulation parame-ters, urinalysis, urine culture, and kidney-ureter--bladder (KUB) radiography were collected from all patients before the procedure. For the purpose of determining stone characteristics, skin-stone‑bladder (KUB) radiography were collected from all patients before the procedure. For the purpose of determining stone characteristics, skin‑stone distance, surrounding anatomy, and urinary tract abnormality, noncontrast computed tomography (CT) was routinely performed.

**TABLE 1:** Patient characteristics

Parameter		Group 1 (n = 79)	Group 2 (n = 79)	P-value
Age, y, mean (SD)		44.6 (12.4)	44.2 (13.7)	0.87
Sex, n (%)	Men	53 (67.1)	48 (60.8)	0.41
	Women	26 (32.9)	31 (39.2)	
BMI, kg/m², mean (SD)		27.4 (3.5)	26.6 (4.3)	0.23
Laterality, n (%)	Right	40 (50.6)	47 (59.5)	0.26
	Left	39 (49.4)	32 (40.5)	
Stone volume, mm³, median (IQR)		431.2 (241.6–805.4)	381.3 (225.9–691)	0.3
Stone density, HU, mean (SD)		823.1 (238.7)	806.4 (215)	0.65
Hydronephrosis, n (%)	0	15 (19)	12 (15.2)	0.53
	I	33 (41.8)	32 (40.5)	
	II	26 (32.9)	25 (31.6)	
	III	4 (5.1)	9 (11.4)	
	IV	1 (1.3)	1 (1.3)	
Skin-to-stone distance, mm, mean (SD)		101.4 (17.9)	100.3 (19.5)	0.71

Abbreviations: BMI, body mass index; HU, Hounsfield unit; IQR, interquartile range

**TABLE 2:** Procedural characteristics and complications

Parameter		Group 1 (n = 79)	Group 2 (n = 79)	P value
Fluoroscopy time, s, mean (SD)		217 (90)	117 (37)	0.001
Number of sessions, mean (SD)		2.18 (0.86)	2.08 (0.87)	0.4
Number of shockwaves, mean (SD)		6311.4 (2478.4)	6069 (2544.6)	0.38
Stone-free status, n (%)		51 (64.6)	53 (67)	0.87
Auxiliary procedures				
None, n (%)		64 (81)	69 (87.3)	0.52
JJ stent insertion, n (%)		7 (8.9)	4 (5.1)	
Stone surgery, n (%)		8 (10.1)	6 (7.6)	
Complications				
None, n (%)		63 (79.8)	65 (82.2)	0.69
Steinstrasse formation, n (%)		12 (15.2)	12 (15.2)	
Pyelonephritis, n (%)		2 (2.5)	1 (1.3)	
Other, n (%)		2 (2.5)	1 (1.3)	
Clavien-Dindo classification of complications, n (%)	Grade 1	26 (32.9)	29 (36.7)	
	Grade 2	12 (15.2)	11 (13.9)	
	Grade 3	8 (10.1)	6 (7.5)	

P values <0.05 were considered significant.

Patients with anatomical anomalies, coagulation disorders, nonopaque calculi, active urinary tract infections, and distal obstruction were excluded from the study. General anesthesia was not administered. The patients were subjected to a maximum of 3 SWL sessions performed by the same physician and technician, both of whom had over 10 years of experience. The interval between the sessions was 1 week, and the patients with residues visible on KUB radiography before the session were included in the next one. Analgesia was provided by intramuscular administration of diclofenac sodium 75 mg 30 minutes before the procedure. During each session, a maximum of 3000 shocks were administered at 90 shocks/minute, using 1–6 energy levels (10–16 kV). The intervention began with an energy level of 1 and 60 shocks/minute frequency. According to the patient’s tolerance, the frequency was increased to 90 shocks/minute, and the energy level was incremented to 6. Administration of shocks was ceased when no residual stone fragments were observed on fluoroscopic evaluation. Fluoroscopy time was recorded at the end of each session. Direct radiography and ultrasound of the urinary tract were performed at the end of the first month after treatment completion. The patients with stones smaller than 4 mm were considered stone-free. All complications were recorded according to the Clavien–Dindo classification (grade 1, colic pain, transient hematuria; grade 2, steinstrasse treated with hydration or α blockers, urinary tract infection, blood transfusion, and perirenal hematoma; grade 3, steinstrasse treated with JJ stent placement / SWL / ureteroscopy / percutaneous nephrostomy, renal hematoma, or hematuria needing intervention; grade 4, neighboring organ injury / hemoptysis, septic shock; grade 5, death).^10^**^,^**^11^

### Statistical analysis

Descriptive statistics were used to describe variables using mean with SD or frequency and percentage. The distribution of variables was checked using the Kolmogorov–Smirnov test. Quantitative data were compared using the independent samples *t* test and the Mann–Whitney test. Qualitative data were compared using the χ^2^ test. The effect level was investigated using univariate and multivariate logistic regression. Statistical analyses were performed using SPSS 26.0 (IBM Corp., Armonk, New York,United States).

### Ethics

All participants were informed that their data would be used for clinical research purposes and gave their written informed consent to have the data recorded in a private database. This study was conducted in compliance with the International Standard for Good Clinical Practice and the principles of the Declaration of Helsinki. The Ethics Committee of the Kartal Dr.Lütfi Kirdar City Hospital in Istanbul approved this study (2020/514/182/6; ClinicalTrials.gov, NCT06689683).

## RESULTS 

The study population comprised 170 patients, 5 of whom were excluded due to proce‑ dure intolerance, and 7 were lost to follow‑up. Fi‑ nally, a total of 158 patients were examined for kidney stone–free status 1 month after the procedure.TABLE 1 shows the demographic data and stone parameters of the patients. The groups were similar in terms of age (P = 0.87), sex (P = 0.41), laterality (P = 0.26), and body mass index (BMI; P = 0.23). The mean (SD) stone volume was 584.3 (450) Hounsfield units in group 1 and 503.6 (376.7) in group 2 (P = 0.3). There was no differ‑ ence in stone density between the groups (mean [SD] 823.1 [238.7] in group 1 vs 806.4 [215] in group 2; P = 0.65). The groups were also similar in terms of hydronephrosis (P = 0.53) and skin‑to‑stone distance (P = 0.71). TABLE 2 shows the SWL data, complication information, and additional intervention data.

Fluoroscopy duration in group 1 was 217.09 (90.2) seconds, while in group 2, it was 117.2 (37.3) seconds (P <0.001). The mean number of sessions (P = 0.4) and shock waves (P = 0.38) was similar in both groups. A total of 51 patients (64%) in group 1 and 53 patients (67%) in group 2 were found to be stone‑free. There was no dif‑ ference between the groups (P = 0.87) in terms of stone‑free rates. Grade 1 complications, such as renal colic and hematuria, were observed in 26 patients (32.9%) in group 1 and 29 patients (36.7%) in group 2. In each group, 1 individual was identified as having grade 2 renal hematoma that did not require intervention. JJ stent insertion for steinstrasse was required in 4 patients from group 1 and 2 patients from group 2. No inter‑ group differences were observed regarding auxiliary interventions (P = 0.52) or overall complications (P = 0.69; TABLE 2 .

## DISCUSSION 

This study aimed to evaluate the possibility of reducing radiation exposure of patients and practitioners during SWL sessions, by extending the intervals of fluoroscopy controls. Our investigation demonstrated that extending the control interval to 500 shots yielded favorable outcomes and significantly reduced fluoroscopic exposure time, without affecting stone-free status.

Imaging modalities that involve radiation are frequently used for diagnosis, treatment, and follow-up. Although commonly used, CT scans, KUB radiography, and intravenous urography expose patients to radiation.^12^ The International Commission on Radiological Protection sets safe exposure levels at 50 milisieverts (mSv) per 1 year or 20 mSv per year averaged over 5 years.^13^ According to previous studies,^14^**^,^**^15^ 20% of patients with nephrolithiasis were exposed to more than 50 mSv of radiation in their first year of treatment. The risk of exceeding the radiation dose limit is higher in patients with multiple stones. The average radiation dose received during SWL is 1.6 mSv, depending on fluoroscopy duration.^15^**^,^**^16^ It is well established that excessive doses of radiation can cause cancer.^15^**^,^**^17^ Therefore, achieving favorable outcomes while minimizing radiation exposure is of great importance.

Radiation dose is influenced by various factors, such as fluoroscopy time, source-to-skin distance, C-arm settings, BMI, projection angle, and target density.^18^ The most common method of assessing the radiation dose received by a patient during a radiological examination is through entrance surface dose (ESD) measurement. To measure ESD, dosimeters are placed on the patient at the entrance surfaces of the X-ray beam.^18^**^,^**^19^ Due to financial and technical reasons, we were not able to measure radiation exposure of the patients with dosimeters. Therefore, we employed fluoroscopy time as a reference for the radiation dose received. Prior research suggests that total fluoroscopic time serves as a critical indicator of radiation exposure in fluoroscopy.^20^

Excessive use of fluoroscopic control might lead to higher radiation exposure for both the patient and medical personnel. Treatment and follow-up strategies should aim to minimize it. There is concern that reducing the frequency of fluoroscopic controls might increase the probability of procedure failure due to inadequate focus on the stone. However, our study demonstrated similar outcomes with shorter control intervals. Standard fluoroscopy devices operate at a frequency of 30 frames per second. However, contemporary models are equipped with a pulsed fluoroscopy mode, enabling a reduction to 1 frame per second.^20^ Elkoushy et al^21^ compared standard fluoroscopy with pulsed fluoroscopy in ureteroscopy and percutaneous nephrolithotomy. Both groups demonstrated similar success rates and reduced fluoroscopy time. However, pulsed fluoroscopy can lead to challenges in achieving sufficient focusing in instances with substantial respiratory movement. Therefore, continuous fluoroscopy may be required in these situations.^22^ We performed SWL without using sedative anesthesia, which is why pulsed fluoroscopy was unfeasible due to respiratory motion.

Various studies on SWL with variable fluoroscopic control intervals reported favorable outcomes. In their research on SWL, Pace et al^23^ and Honey et al^24^ performed fluoroscopic control every 200 shocks. In a study by Besien et al^6^ comparing ultrasound-guided and fluoroscopic SWL, position adjustment was performed every 300 shocks, with an average fluoroscopy time of 178 seconds. A similar study comparing ultrasound-guided and fluoroscopic SWL utilized fluoroscopic control every 600 shocks, but no information was provided on the average fluoroscopy time.^4^ The studies mentioned above reported favorable outcomes in terms of stone-free rates using different control intervals. However, there is no study comparing the effect of different control intervals on the stone-free rate. At our center, fluoroscopic control is routinely performed every 250 shocks. Our study involved 2 groups that received fluoroscopic control every 250 and 500 shocks, respectively. Both groups showed comparable stone-free rates. However, an extended control interval (every 500 shocks) was associated with significantly lower fluoroscopy duration, in the result of which group 2 experienced lower radiation exposure.

There are several factors affecting fluoroscopy duration, including the practitioner’s experience.^25^^,^^26^^,^^27^ Ordon et al^25^ found that the practitioner’s experience did not impact stone-free status. They reported that patients with longer total fluoroscopy duration were more likely to achieve a stone-free status. In contrast, a study comparing the outcomes of 6 different radiology technicians concluded that the most experienced technicians achieved the highest stone-free status rate in the shortest time.^26^ In our investigation, all SWL procedures were performed by the same technician and physician, and both had more than 10 years of expertise. Thus, we eliminated the impact of the practitioner’s experience. However, we observed a comparable stone-free rate in the group with longer fluoroscopy duration. Nevertheless, since the control intervals were not identical in our study, we cannot conclude whether the duration of fluoroscopy affected the stone-free status.

Our research demonstrated that favorable results could be achieved with longer intervals between fluoroscopic controls. However, it has certain limitations. We did not quantify the actual radiation dose received by the patients. In addition, our research was limited to renal pelvic stones. Thus, findings regarding stones located in other regions of the kidney or those located in the ureter may differ, which warrants further studies.

## CONCLUSIONS 

This prospective randomized study demonstrated that fluoroscopic control could be performed every 500 shocks during an SWL session without affecting the stone-free status. Our study showed favorable results with decreased radiation exposure. Standardizing the fluoroscopy interval during SWL is essen-tial to prevent unnecessary radiation exposure.

## JOURNAL INFORMATION

Videosurgery and Other Miniinvasive Techniques is an official journal of the Videosurgery Foundation.
